# Endovascular management of a mycotic group A streptococcal abdominal aortic dissection

**DOI:** 10.1259/bjrcr.20150332

**Published:** 2016-07-27

**Authors:** John Colville, Manmohan Madan, Khalid Bashaeb, Riza Ibrahim, Abysinia Sibanda

**Affiliations:** ^1^Radiology, Royal Oldham Hospital, Pennine Acute Hospitals NHS Trust, Oldham, UK; ^2^Vascular Surgery, Royal Oldham Hospital, Pennine Acute Hospitals NHS Trust, Oldham, UK

## Abstract

Pyrexia of unknown origin can represent a great diagnostic difficulty to clinicians. We present a case of pyrexia with abdominal and back pain, in which blood cultures performed demonstrated group A haemolytic streptococcus. Having recently been abroad, the patient was investigated to find a source. CT scans performed subsequently demonstrated an inflammatory infrarenal abdominal aortic dissection. The patient was treated with intravenous antibiotics and underwent endovascular repair. This case details the unusual presentation of spontaneous abdominal aortic dissection and its management by endovascular means. Emphasis is placed on the often clandestine manner of presentation of this condition and the importance of awareness in the investigating clinician. This case presents a patient infected with group A haemolytic streptococcus, leading to aortitis and spontaneous dissection, previously unreported in the literature.

## Clinical presentation and early investigations

A 58-year-old male presented to the accident and emergency department with pyrexia of unknown origin, complaining of feeling feverish for the past 4 days. He had been vomiting excessively, had reduced oral intake and complained of a cough of around 2 weeks duration. He had returned from Kashmir 6 weeks prior to presentation and had a past medical history of malarial infection, hypertension and hypercholesterolaemia. He was noted to be clammy and feverish on examination, with right-sided abdominal and back pain. On arrival, his vital signs were: temperature 39°C, blood pressure 139/89 mmHg, heart rate 85 beats per minute, respiratory rate 18 breaths per minute and oxygen saturation 94%.

The patient was empirically started on broad-spectrum antibiotics tazocin and clarithromycin, while investigations took place. A diagnosis of typhoid or malaria was suspected owing to his recent travel. Chest X-ray, malaria parasite screen, human immunodeficiency virus, hepatitis B and C serology, atypical pneumonia screen, urine testing for legionella, and pneumococcal and viral throat swabs, all produced negative results. Peripheral blood cultures taken on presentation grew group A streptococcus, the source of which was unclear.

On admission, haemoglobin was 12.1 g dl^–1^ (normal 13–18); white blood cell count 6.9 × 10^9^ l^–1^ (normal 4.0–11.9); platelets 145 × 10^9^ l^–1^ (normal 150–450); sodium 136 mmol l^–1^ (normal 136–145); potassium 3.4 mmol l^–1^ (normal 3.5–5.4); creatinine 84 mmol l^–1^ (normal 62–115); urea 4.4 mmol l^–1^ (normal 2.5–6.7); eGFR 79 ml min^–1^. C-reactive protein levels rose from 64.5 on admission (normal <10 mg l^–1^) to a peak of 355.8 mg l^–1^ on day 3 of admission. In addition, liver function tests became impaired: from normal levels at admission, bilirubin rose to 47 umol l^–1^ (normal 3–21); alanine transaminase to 190 u l^–1^ (normal 10–35); alkaline phosphatase to 462 u l^–1^ (normal 30–150); albumin fell to 21 g l^–1^ at its lowest (normal 35–50). International normalized ratio was mildly deranged at 1.3 (normal 0.9–1.1). Consequently, a differential diagnosis of biliary sepsis was considered.

## Radiological investigation

Abdominal ultrasound was performed to assess for biliary obstruction. A single calculus was noted within the gallbladder; however, no duct dilatation was found and there was no evidence of inflammatory change in the gallbladder. The pancreas was described as hyperechoic. The aorta was commented to be 3 cm in diameter. Further investigation with venous phase CT scan of the pancreas was obtained on day 4, which did not demonstrate evidence of inflammatory change or infection in the gallbladder or pancreas to explain the patient’s presentation. Additionally, an MR cholangiopancreatography examination was performed, which showed no evidence of common bile duct dilatation, cholecystitis or choledocolithiasis.

**Figure 1.**
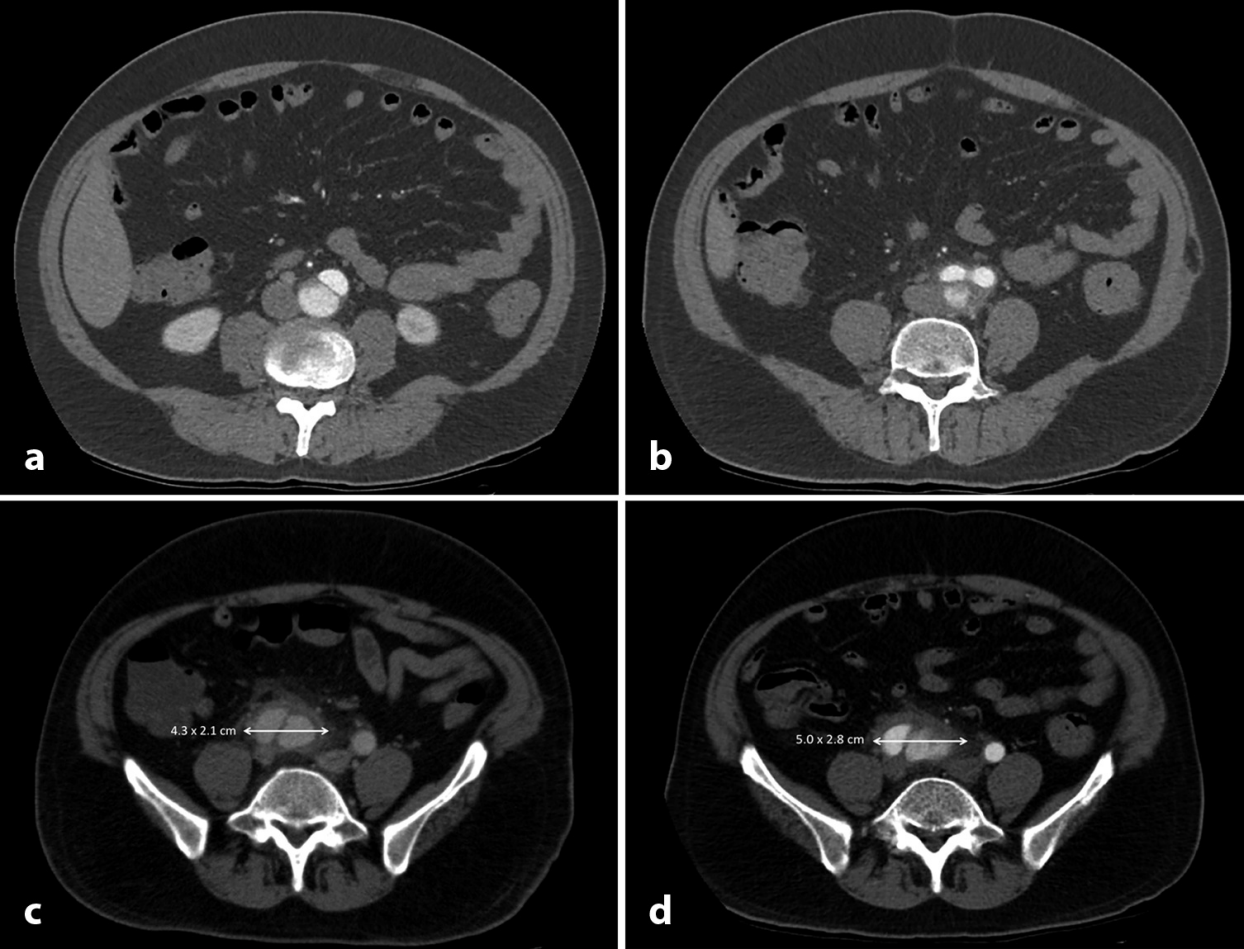
Pre-operative arterial phase (a, b and d) and venous phase (c) multidetector CT scans with 0.1 cm slices through the abdomen and pelvis. Axial images show the dissection arising in the infrarenal aorta (a) and extending across the aortic bifurcation (b) to the right internal iliac artery (c and d). Periaortic fat stranding indicates indolent infection and inflammation. Despite conservative management, persistence of inflammatory changes could be seen with an increase in the size of the right common iliac aneurysmal sac from 4.3 × 2.1 cm (c) to 5.0 × 2.8 cm (d) 6 days later. Such rapid aneurysmal dilatation seen in less than 1 week indicated that, if left untreated, the aneurysm was at risk of further expansion and rupture.

However, the CT scan revealed there to be a dissecting abdominal aortic aneurysm, with the false lumen arising distal to the level of the renal arteries and extending just proximal to the bifurcation of the right common iliac artery. The appearance was that of a mycotic aneurysm, with the walls of the aorta appearing inflamed with periaortic and peri-iliac fat stranding present (Figure 1). Given the clinical picture of right-sided abdominal and back pain with an acute inflammatory response, the radiological findings of aortitis and the absence of other causative pathology, it was agreed that the dissection represented the source of the patient’s sepsis.

A subsequent arterial phase CT scan performed on day 10 of admission showed that despite medical management, there was rapid progression of the right common iliac aneurysm (Figure 1d), raising concerns of impending rupture if left untreated.

## Treatment

The clinical and radiological findings were discussed at the vascular multidisciplinary meeting. A robust evidence-based discussion on endovascular and surgical treatment options against conservative management took place. The patient was a young, fit gentleman with an abdominal aortic dissecting aneurysm extending just proximal to the bifurcation of the right common iliac artery. Open replacement of the aorta and iliac arteries was considered, which would require harvesting of saphenofemoral veins bilaterally, carrying a significant mortality and morbidity risk such as ischaemic bowel, deep vein thrombosis and impotence. The final decision to treat by endovascular means was reached after discussions involving the patient.

Prior to the insertion of a covered stent, it was essential to treat the sepsis, as any contamination would later require open repair. Following discussions with the microbiology department, the patient was treated with teicoplanin, meropenem and gentamicin according to sensitivities. When serial blood cultures showed no further growth and an echocardiogram displayed no cardiac source, the patient was prepared for treatment.

**Figure 2.**
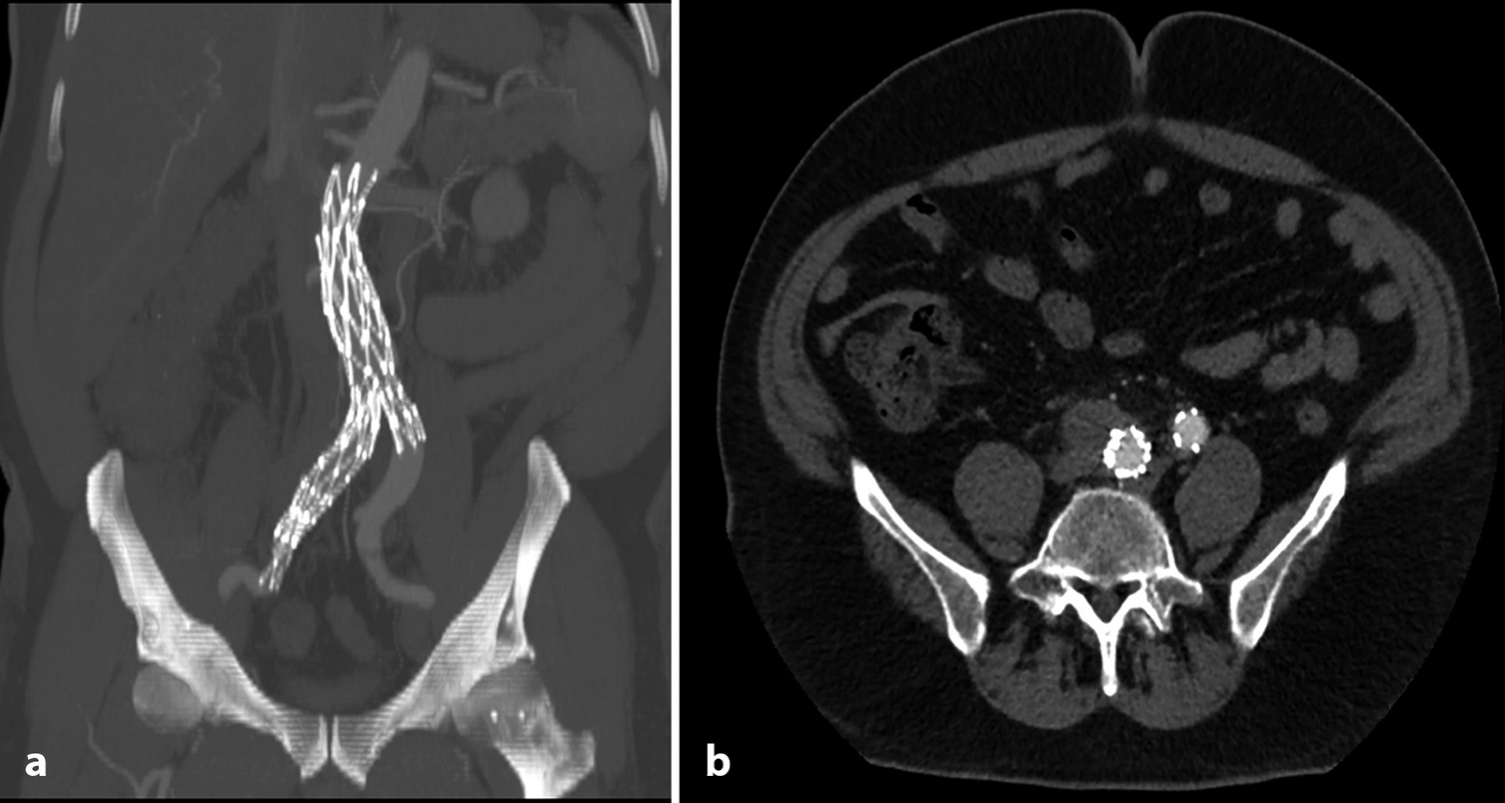
CT surveillance scan performed 3 months after treatment shows the position of the endograft in the aorta and the right common iIiac artery (a). Arterial phase axial image demonstrates complete resolution of inflammatory changes and significant reduction in the size of the aneurysm sac to 3.7 × 2.5 cm (b).

Endovascular repair was carried out on day 12 of admission with a bifurcated AFX endograft (Endologix, Irvine, CA). This device was used owing to the narrow aortic bifurcation (Figure 2a), with anatomical fixation on the aortic bifurcation to obliterate the false lumen. The right internal iliac artery was embolized using coils prior to stenting to avoid endoleak. The endograft was extended into the right external iliac artery to exclude the right common iliac artery aneurysm.

## Outcome and follow-up

The patient was well postoperatively and was discharged with 6-month oral antibiotic courses of erythromycin and ciprofloxacin on day 15 of admission. Repeat blood tests performed at 2 months showed a return to normal parameters apart from a mildly elevated C-reactive protein (15 mg l^–1^). The patient has been followed up for 2 years postoperatively. Follow-up CT angiography at 3 months showed a patent endograft in a satisfactory position, with no endoleaks or aneurysmal dilatation (Figure 2a). Furthermore, there was resolution of the inflammatory changes and complete exclusion of the aneurysm (Figure 2b). Yearly follow-up has shown no subsequent graft infection, complications or requirement for secondary interventions. The patient is now being followed up annually in the clinic with arterial duplex examination.

## Discussion

Aortic dissection arises from infiltration of blood into the tunica media, where it divides the aortic wall into an inner segment comprising the intima and part of the media, and an outer segment, which comprises part of the media and the adventitia. The consequence is the formation of a double lumen aorta with a true and a false lumen.

Hypertension has been shown to be the greatest risk factor in the formation of dissection. Other causes include collagen disorders such as Marfan and Ehlers–Danlos syndromes, coarctation of aorta, bicuspid aortic valve, hypoplasia of aorta, cystic medial necrosis, trauma, pregnancy, iatrogenic causes and aortitis.

Aortitis can cause aneurysm, dissection and rupture, leading to cardiopulmonary collapse. The majority of aortitis is due to non-infective vasculitides such as giant cell arteritis, Takayasu arteritis, rheumatoid arthritis, systemic lupus erythematosus and human leukocyte antigen B27-associated spondyloarthropathies, among others. Giant cell arteritis is the most common cause of non-atherosclerotic aortitis, accounting for 72.7% of cases,^12^ while infective aortitis is a relatively uncommon finding, representing 16.7% of non-atherosclerotic aortitis and 2.6% of abdominal aortic aneurysms.^13^ Case series show *Staphylococcus aureus* (30%) and salmonella species (50%) are the predominant organisms in mycotic aneurysms in the post-antibiotic era.^14^ However, recently, the rise of other causative organisms such as *Streptococcus pneumoniae* (33%) have been identified in the literature. Other rarer pathogens such as *Listeria monocytogenes, Clostridium septicum, Streptococcus milleri* and group B streptococcus have also been reported.^1–7^ We have presented a case of aortitis secondary to group A haemolytic streptococcus, which, to the best of our knowledge, has not been previously reported in the literature.

Spontaneous isolated infrarenal abdominal aortic dissection is very rare (around 2.5% of dissection presentations) and clinicians are unfamiliar with its presentation, as its symptoms are often non-specific. Furthermore, presentation is often clandestine and can mimic other conditions, resulting in an inevitable and understandable delay in reaching a diagnosis. Previous case reports describe patients being referred to urology as suspected renal colic and retention before subsequent CT investigation was able to elicit aortic pathology.^8,9^ In others, the presenting complaint was of lower limb and buttock claudication and cases of spinal cord ischaemia resulting in lower limb paresthesia and paraplegia.^10,11^ Indeed, the presentation is varied and may be inconsistent with the site of pathology.

The complexity of this case inevitably led to a delay in diagnosis. The patient was initially managed as sepsis of unknown origin under the care of the medical team, while efforts focussed on excluding conditions such as malaria and atypical pneumonia. This approach was appropriate given the patient’s recent travel and past medical history. Furthermore, the symptoms of right-sided abdominal and back pain are not specific to aortic pathology. In addition to gradually worsening liver function tests (thought to represent the effect of systemic sepsis in combination with broad-spectrum antibiotics such as tazocin), it was sensible to consider biliary sepsis as a differential diagnosis until it was excluded on MR cholangiopancreatography examination. The combination of clinical, laboratory and radiological findings led to the diagnosis of a mycotic abdominal aortic dissecting aneurysm secondary to group A haemolytic streptococcus. Unfortunately, as a result of the endovascular procedure, it was not possible to sample the diseased vessel wall for confirmation. However, clinical improvement in the patient’s condition, and the resolution of biochemical markers of sepsis and radiographic features of inflammation at 3 months strongly supports this diagnosis. Consequently, early investigation, a broad differential diagnosis and involvement of other specialties are essential in the management of pyrexia of unknown origin.

Management of mycotic dissection is fraught with technical difficulties and involves a combination of antibiotic therapy and surgical management. Aortic reconstruction can be performed by open surgical methods involving the following: aneurysm excision, debridement, aortic oversewing and extra-anatomic bypass. However, owing to reportedly high mortality rates (up to 36%) and complications such as stump blowout and graft occlusion, often *in situ* reconstruction is preferred. *In situ* autologous vein reconstruction using harvested superficial veins, such as the great saphenous vein, is a durable treatment option. In some cases, antibiotic-impregnated or silver-coated synthetic grafts and cryopreserved homografts can provide treatment alternatives.^15^

When introducing a synthetic graft into the aorta, infective organisms must be eradicated to avoid post-operative complications secondary to infection. Despite this, one study reported 90% survival at 30 days and 82% at 2 years for mycotic aortic aneurysms treated by endovascular means.^16^ However, late complications due to infection can occur and often prove fatal, with one multicentre study reporting 27% reinfection and 70% mortality rates for those affected.^17^ It was therefore vital to ensure that the patient was discharged with long-term oral antibiotics to help prevent such complications. In fact, the same study was able to show favourable 5-year survival data of 55% for mycotic aneurysms managed by endovascular means compared with 35% in a similar study that looked at open repair.^18^ Furthermore, the patient presented in this case has remained well, with no recurrence of infection at 24 months follow-up. This case therefore demonstrates that it is possible to safely manage mycotic abdominal aortic dissecting aneurysms by endovascular means, following targeted pre-operative and post-operative antibiotic therapy.

The case highlights both diagnostic and treatment challenges faced by clinicians when treating cases presenting with abdominal aortic dissection secondary to group A streptococcus aortitis presenting as pyrexia of unknown origin.

## Learning points

1. Spontaneous infrarenal abdominal aortic dissection can present in a myriad of different ways, often in a clandestine fashion. Clinicians need to maintain a high index of suspicion when investigating patients with pyrexia of unknown origin.
2. We present a rare case of group A haemolytic streptococcus aortitis, leading to formation of dissection.
3. We present how after targeted antibiotic therapy, it is possible to treat mycotic dissections by endovascular methods.

## Consent

Informed consent to publish this case was obtained and is held on record
